# Evaluation of [^18^F]F-DPA PET for Detecting Microglial Activation in the Spinal Cord of a Rat Model of Neuropathic Pain

**DOI:** 10.1007/s11307-022-01713-5

**Published:** 2022-03-18

**Authors:** Saeka Shimochi, Thomas Keller, Ella Kujala, Joonas Khabbal, Johan Rajander, Eliisa Löyttyniemi, Olof Solin, Pirjo Nuutila, Shigehiko Kanaya, Emrah Yatkin, Tove J. Grönroos, Hidehiro Iida

**Affiliations:** 1 Turku PET Centre, University of Turku, Turku, Finland; 2 Medicity Research Laboratory, University of Turku, Turku, Finland; 3 Nara Institute of Science and Technology, Ikoma City, Japan; 4 Central Animal Laboratory, University of Turku, Turku, Finland; 5 Accelerator Laboratory, Turku PET Centre, Åbo Akademi University, Turku, Finland; 6 Department of Biostatistics, University of Turku, Turku, Finland; 7 Turku PET Centre, Turku University Hospital, Turku, Finland

**Keywords:** Neuropathic pain, Microglia, Spinal cord, Translocator protein 18 kDa (TSPO), Positron emission tomography (PET), [^18^F]F-DPA, [^11^C]PK11195

## Abstract

**Purpose:**

Recent studies have linked activated spinal glia to neuropathic pain. Here, using a positron emission tomography (PET) scanner with high spatial resolution and sensitivity, we evaluated the feasibility and sensitivity of N,N-diethyl-2-(2-(4-([^18^F]fluoro)phenyl)-5,7-dimethylpyrazolo[1,5-a] pyrimidin-3-yl)acetamide ([^18^F]F-DPA) imaging for detecting spinal cord microglial activation after partial sciatic nerve ligation (PSNL) in rats.

**Procedures:**

Neuropathic pain was induced in rats (*n* = 20) by PSNL, and pain sensation tests were conducted before surgery and 3 and 7 days post-injury. On day 7, *in vivo* PET imaging and *ex vivo* autoradiography were performed using [^18^F]F-DPA or [^11^C]PK11195. *Ex vivo* biodistribution and PET imaging of the removed spinal cord were carried out with [^18^F]F-DPA. Sham-operated and PK11195-pretreated animals were also examined.

**Results:**

Mechanical allodynia was confirmed in the PSNL rats from day 3 through day 7. *Ex vivo* autoradiography showed a higher lesion-to-background uptake with [^18^F]F-DPA compared with [^11^C]PK11195. *Ex vivo* PET imaging of the removed spinal cord showed [^18^F]F-DPA accumulation in the inflammation site, which was immunohistochemically confirmed to coincide with microglia activation. Pretreatment with PK11195 eliminated the uptake. The SUV values of *in vivo* [^18^F]F-DPA and [^11^C]PK11195 PET were not significantly increased in the lesion compared with the reference region, and were fivefold higher than the values obtained from the *ex vivo* data. *Ex vivo* biodistribution revealed a twofold higher [^18^F]F-DPA uptake in the vertebral body compared to that seen in the bone from the skull.

**Conclusions:**

[^18^F]F-DPA aided visualization of the spinal cord inflammation site in PSNL rats on *ex vivo* autoradiography and was superior to [^11^C]PK11195. *In vivo* [^18^F]F-DPA PET did not allow for visualization of tracer accumulation even using a high-spatial-resolution PET scanner. The main reason for this result was due to insufficient SUVs in the spinal cord region as compared with the background noise, in addition to a spillover from the vertebral body.

## Introduction

Neuropathic pain is a chronic condition that occurs after nerve damage from, *e.g.*, cancer, infection, autoimmune disease, trauma, and diabetes. Peripheral nerve injury can lead to neuropathic pain followed by allodynia (pain from normally innocuous stimuli) and hyperalgesia (exaggerated response to noxious stimuli) [1, 2]. Although unraveling the mechanisms of pain hypersensitivity resulting from nerve damage is essential to developing new therapeutic drugs for neuropathic pain, the mechanism of pain onset remains unknown. Conventional nonsteroidal anti-inflammatory drugs and narcotic analgesics do not work, often leaving people with a reduced quality of life [3, 4]. Since pain is a subjective expression of a pathological condition with large individual differences, a robust biomarker and a diagnostic tool that could accurately identify neuropathic pain, and treatment responses, would be highly valuable.

Despite the limited understanding of the mechanisms, recent findings have implicated activated spinal glia [5, 6]. These cells have been reported to release several types of pain-inducing factors, and the degree of microglial activation in the dorsal horn correlates with allodynia severity, suggesting that microglial activation in the spinal dorsal horn could be pivotal in the development and progression of neuropathic pain [7].

Neuroinflammation is associated with increased expression of the 18-kDa translocator protein (TSPO) of activated microglia. *In vivo* positron emission tomography (PET) imaging of neuroinflammation attributed to TSPO expression is an attractive method for evaluating disease progression and therapeutic outcomes [8, 9]. The non-invasiveness of this method enables repetitive measurements on individuals, which allows for longitudinal assessment during disease progression and after the therapeutic intervention. Imamoto et al. [10] reported that glial activation in the spinal cord could be quantitatively imaged in a rat model of neuropathic pain, the partial sciatic nerve ligation (PSNL) model, using the TSPO radioligand (R)-N-methyl-[^11^C]-1-(2-chlorophenyl)-N-(1-methylpropyl)-3-isoquinolinecarboxamide ([^11^C]PK11195). Recently, a novel F-18–labeled TSPO-specific radioligand, N,N-diethyl-2-(2-(4-([^18^F]fluoro)phenyl)-5,7-dimethylpyrazolo[1,5-a] pyrimidin-3-yl)acetamide ([^18^F]F-DPA), was developed at the Turku PET Centre [11–16]. The main difference between the molecular structures of [^18^F]F-DPA (*K*_*i*_ = 1.7 nM) and [^18^F]DPA-714 (*K*_*i*_ = 7.0 nM) is that the fluorine atom is directly linked to the phenyl moiety in [^18^F]F-DPA, whereas a spacer chain is needed in [^18^F]DPA-714 [11]. [^18^F]F-DPA has a much greater binding affinity for TSPO than [^18^F]DPA-714 or [^11^C]PK11195 (*K*_*i*_ = 3.6 nM), which is advantageous in imaging a small lesion. The longer physical half-life (*T*_½_ = 110 min) and the lower positron range of F-18 compared with that of C-11 (*T*_½_ = 20 min) are also advantageous.

PET imaging of the spinal cord of small experimental animals is challenging because of the small target size, particularly when the surrounding vertebral column might accumulate the radiopharmaceutical. A commercially available state-of-the-art PET scanner, β-Cube (Molecubes, Ghent, Belgium), provides a spatial resolution < 1.0 mm [17, 18] that could enable imaging of inflammation in small animal spinal cords. Thus, we hypothesized that the use of a high-resolution and high-sensitivity PET scanner with [^18^F]F-DPA allows for reliable identification of the glial activation in the spinal cord in a rat PSNL model of neuropathic pain.

In the present study, using a PET scanner with high spatial resolution and sensitivity, we aimed to evaluate the feasibility and sensitivity of the [^18^F]F-DPA PET imaging technique for detecting spinal cord microglial activation in a PSNL model of neuropathic pain induced by peripheral nerve injury.

## Material and Methods

### Animal Model of Neuropathic Pain

**Fig. 1.**
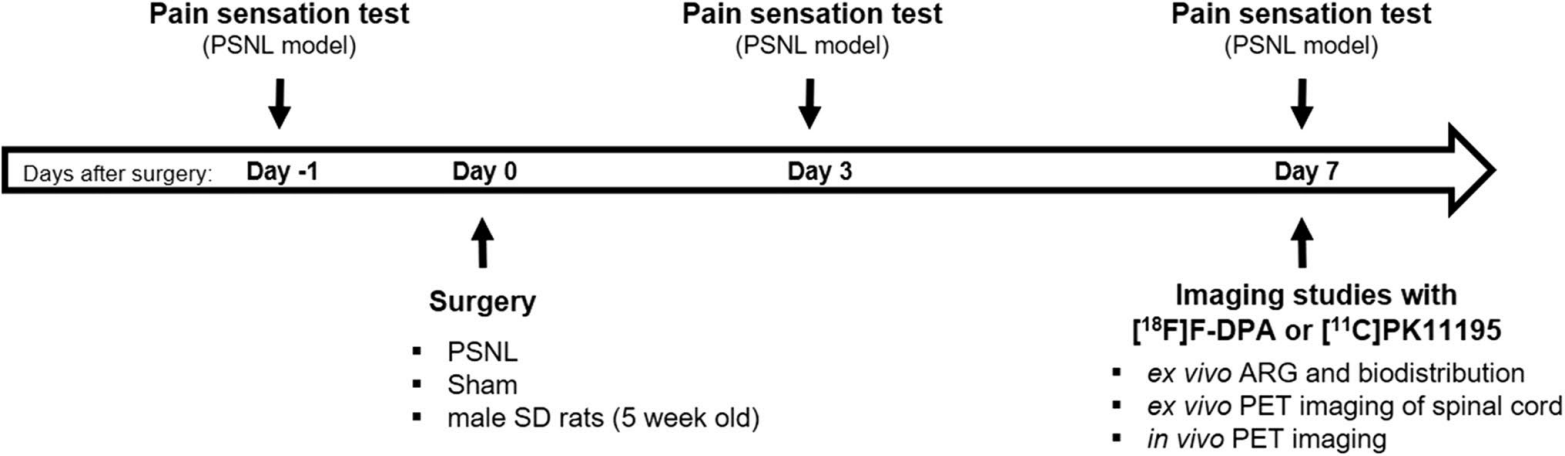
Schedule and set-up of the experiments performed in this study. PSNL, partial sciatic nerve ligation; ARG, autoradiography

Animals were group-housed under standard conditions under a 12-h light–dark cycle in controlled conditions (temperature 21 ± 3 °C, humidity 55 ± 15%) at the Central Animal Laboratory, University of Turku, and had ad libitum access to RM3(E) rodent diet (SDS, UK) and tap water. The study design is illustrated in Fig. 1, and the number of animals used in each experimental set-up is presented in Table 1.

**Table 1.** The numbers of animals, weights, and amounts of injected doses used in the study. Data are expressed as mean ± SD

	Animal number (*n*)	Animal model	Animal weight (g)	Injected dose (MBq)	Injected mass (µg)	*A*_m_at time of inj. (GBq/µmol)
PET imaging						
[^18^F]F-DPA Molecubes	3	PSNL	145 ± 10	8.9 ± 1.3	1.6 ± 0.6	2.2 ± 1.1
[^18^F]F-DPA Inveon	3	PSNL	166 ± 3	49.6 ± 2.0	5.2 ± 1.4	3.6 ± 0.9
[^11^C]PK11195 Molecubes	3	PSNL	155 ± 1	10.6 ± 0.7	0.09 ± 0.03	43.4 ± 11.6
*Ex vivo* PET/spinal cord						
[^18^F]F-DPA Molecubes	2	PSNL	158, 149	48.5, 51.4	3.20, 5.80	5.4, 3.1
	2	Sham	219, 219	48.0, 50.8	16.6, 18.0	1.0, 1.0
*Ex vivo* ARG						
[^18^F]F-DPA	6	PSNL	161 ± 8	49.7 ± 1.8	4.9 ± 1.4	3.8 ± 1.1
	2	Sham	219, 219	48.0, 50.8	16.6, 18.0	1.0, 1.0
	1	Pretreated, 1 mg	179	36.8	11.9	1.1
	1	Pretreated, 3 mg	220	48.0	27.7	0.61
[^11^C]PK11195	3	PSNL	148 ± 18	52.1 ± 1.7	0.61 ± 0.31	35.3 ± 15.9

All applicable institutional and/or national guidelines for the care and use of animals were followed. The procedures and protocols were approved by the National Project Authorization Board of Finland in accordance with the Finnish National legislation (Act 497/2013 and Decree 564/2013 on the protection of animals used for scientific or educational purposes) and the EU Directive 2010/EU/63 on the protection of animals used for scientific purposes. The Project license number was ESAVI/20863/2018. The rats were closely monitored for signs of pain and discomfort until the end of the experiment and received analgesics pre- and postoperatively.

To induce chronic neuropathic pain in animals, we used the PSNL model [19], performing surgery in 5-week-old male Sprague–Dawley (SD) rats. The rats were anesthetized with isoflurane (4% for induction and 2% for maintenance). Tight ligation of approximately one-third to one-half of the diameter of the right sciatic nerve (ipsilateral) at the upper-thigh level was performed with a 7–0 silk suture (Ethicon, Somerville, New Jersey). This procedure has been shown to lead to unilateral inflammation in the lumbar spinal cord at levels L3–L6 [10]. In sham animals, the right sciatic nerve was exposed but not ligated. For pain management, buprenorphine (0.3 mg/ml, Temgesic) was administered subcutaneously 30 min before the surgery and twice daily for two consecutive days.

### Pain Sensation Test

A von Frey filament (Ugo Basile, Italy) test was used to evaluate pain sensation in the rats a day before surgery and on days 3 and 7 post-injury. Assessment of the mechanical threshold was done with the up–down method [20]. Rats were placed on a mesh floor covered with an inverted transparent plastic box until exploratory behavior ceased. Nine individual von Frey filaments with forces of 0.4, 0.6, 1.0, 1.4, 2.0, 4.0, 6.0, 8.0, and 10.0 g (Ugo Basile, Italy) were applied to the central region of the plantar surface of the hind paw, and the paw withdrawal threshold (g) was observed. The weakest stimulation that caused the withdrawal response was set as the threshold value.

### Radiochemistry

The electrophilic syntheses of [^18^F]F-DPA were performed according to previously described procedures [11]. The radiochemical purity was greater than 99%. [^11^C]PK11195 was synthesized using the previously published method [21]. Molar activities (*A*_m_) of injected doses are shown in Table 1. The radiochemical purity of the produced [^11^C]PK11195 was greater than 99%, and the molar activity (decay-corrected to time of injection) was 39 ± 13 GBq/μmol.

### *Ex Vivo* Autoradiography

PSNL and sham animals were sacrificed at 40 min ([^18^F]F-DPA) and 30 min ([^11^C]PK11195) post-injection by cardiac puncture under deep anesthesia with 4% isoflurane and perfused transcardially with PBS. Segments of the spinal cord (lumbar at L3–L6 and thoracic at T11-T12) were rapidly removed, frozen in chilled isopentane, and cut on a cryomicrotome (Leica CM3050S) in coronal slices 20 μm ([^18^F]F-DPA) and 40 μm ([^11^C]PK11195) thick. The slides were exposed to an imaging plate (Fuji BAS Imaging Plate TR2025; Fuji Photo Film Co., Ltd.) for approximately two half-lives of the radioisotope and scanned as previously described [11].

The images were analyzed for ^18^F- or ^11^C-radioactivity count densities, which were expressed as background-subtracted photostimulated luminescence per area (PSL/mm^2^). Regions of interest (ROIs) were placed in the ipsilateral and contralateral regions to compare the ipsilateral lumbar spinal cord PSL/mm^2^ values to those for the contralateral lumbar spinal cord (ipsi/contralateral ratio). Additional ROIs were drawn to cover the whole spinal cord regions at the lumbar (inflammation site) and thoracic (reference) levels for comparison of whole lumbar spinal cord PSL/mm^2^ values to those for the thoracic spinal cord (lumbar/thoracic ratio). The thoracic spinal cord and contralateral lumbar spinal cord were used as reference regions.

### Blocking Study

To determine the specificity of the [^18^F]F-DPA binding, 1 mg or 3 mg of PK11195 (ABX GmbH, Radeberg, Germany) was injected intraperitoneally to two PSNL animals 30 min prior to the administration of the tracer. The animals were sacrificed 40 min after the [^18^F]F-DPA injection and the spinal cords were sliced for autoradiography analyses as described above.

### Biodistribution of [^18^F]F-DPA

PSNL animals were sacrificed at 40 min post-injection of [^18^F]F-DPA by cardiac puncture under deep anesthesia with 4% isoflurane and perfused transcardially with PBS. Animals were then dissected, and the blood, spinal cord (lumbar at L3–L6 and thoracic at T11-12), bone from skull, and vertebral body (L1) were harvested for *ex vivo* biodistribution measurements as previously described [11]. The full ex vivo biodistribution data of [^18^F]F-DPA has previously been described [11]. Mean SUVs were calculated for each sample using the same equation as for PET images.

### Immunohistochemical Staining

Microglial and astrocytic activation was assessed using the inflammation markers, Iba1 (1:1000, Wako Ltd., Japan) and GFAP (1:1000, Sigma, Germany) for the fresh-frozen spinal cord slices collected for the autoradiography studies. The immunohistochemistry procedures were performed as described before [22, 23]. Images of the stained slices were taken with a Panoramic 1000 slide scanner (3DHISTECH).

### *Ex Vivo* [^18^F]F-DPA PET Imaging of the Spinal Cord and Data Analysis

Two PSNL and two sham animals were sacrificed at 40 min post-injection of [^18^F]F-DPA and perfused transcardially with phosphate-buffered saline (PBS). The brain and whole spinal cord were rapidly removed and scanned for 10 min (CT) and 60 min (PET) using the Molecubes. VOIs were placed on the lumbar and thoracic spinal cord areas with the same volume for each rat. CT images were used to align the VOIs. Values were corrected for the injected activity and decay. The averaged SUVs inside the VOIs were calculated for each animal, and the SUVs on the highest image pixel in the lumbar and thoracic regions were calculated to assess SUV at the peak. Lumbar/thoracic ratios of each SUV were then calculated.

### *In Vivo* PET/CT Imaging and Data Processing

All PET/CT scans on the PSNL model animals were performed on day 7 after the injury using either Molecubes or Inveon (Siemens, Knoxville, USA) scanners. Following the NEMA guidelines, a detailed comparison of the two PET scanner’s designs and performances has previously been reported from our institution [18].

The rats were kept on a heating pad and under anesthesia with 2% isoflurane/oxygen gas during the study. Immediately after a 10-min CT scan for attenuation correction, a 60-min dynamic PET scan (26 frames: 10 × 2 s, 4 × 10 s, 4 × 60 s, 5 × 300 s, 3 × 600 s) was started simultaneously with the injection of either [^18^F]F-DPA or [^11^C]PK11195 intravenously via a tail vein. The injected dose amounts and masses are presented in Table 1.

PET images were reconstructed using three-dimensional ordered-subsets expectation–maximization (3D OSEM) with a depth-of-iteration compensation in Molecubes, and shifted-Poisson model maximum-a-posteriori (SP-MAP) in Inveon. Carimas 2.1 software (Turku PET Centre) was used to analyze the PET/CT images. CT images were also used to define the location of the spinal cord. Volumes of interest (VOIs), with the same volume for each rat, were placed at levels L3–L6 in the lumbar cord (site for unilateral inflammation) and on the vertebral body in animals. T11-T12 of the spinal cord was used as a reference area throughout the study. Data were corrected for the injected activity and decay, and mean standardized uptake values (SUVs) were calculated for the 40–60-min period.

### Statistical Analysis

Results are presented as mean ± standard deviation (SD). The pain sensation data were analyzed with a linear mixed model for repeated measurements, in which a group was handled as the between factor and time as the within factor. If significance was found, the contrast was programmed into pairwise time point interactions. Ratios were analyzed using an unpaired *t*-test. Multi-way analysis of variance was used to study the effects of the scanner, tracer, and region on SUVs. The assumptions for testing were checked with studentized residuals. The limit for statistical significance was set at *p* < 0.05 (two-tailed). The statistical analyses were performed with SAS software, version 9.4 for Windows (SAS Institute, Cary, NC, USA).

## Results

### Pain Sensation Test

**Fig. 2.**
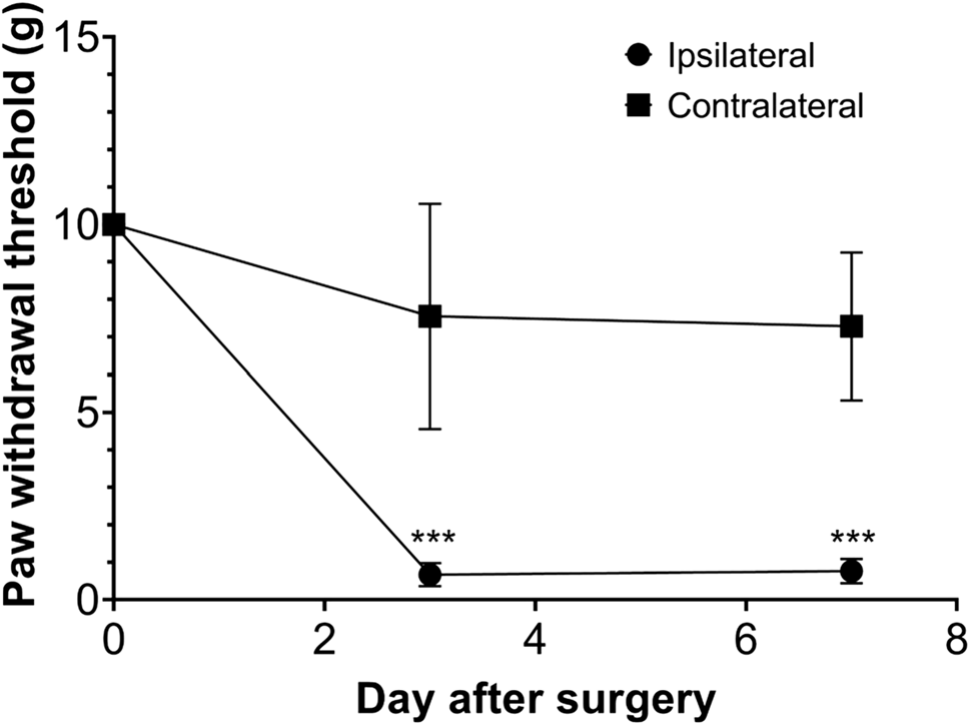
Results from the von Frey filament test used to measure the pain sensation in rats a day before surgery and on days 3 and 7 post-injury. Data are presented as mean ± SD of the paw withdrawal threshold (g). ***p < 0.0001, *n* = 7/group

Partial sciatic ligation of the right sciatic nerve produced mechanical allodynia in the ipsilateral hind paw (Fig. 2). The weakest stimulation that caused the withdrawal response was set as the threshold value. The threshold of the nerve-injured paw decreased from day 3 post-injury and it was constant until day 7. On day 3, the ipsilateral paw responded to stimulation of 0.67 ± 0.31 g, whereas the contralateral paw responded to stimulation of 7.6 ± 3.0 g (*p* < 0.0001). Thereafter, the threshold was constant until day 7 post-injury (0.77 ± 0.33 g vs. 7.3 ± 2.0 g), respectively.

### *Ex Vivo* Spinal Cord Autoradiography and Immunohistochemical Staining

**Fig. 3.**
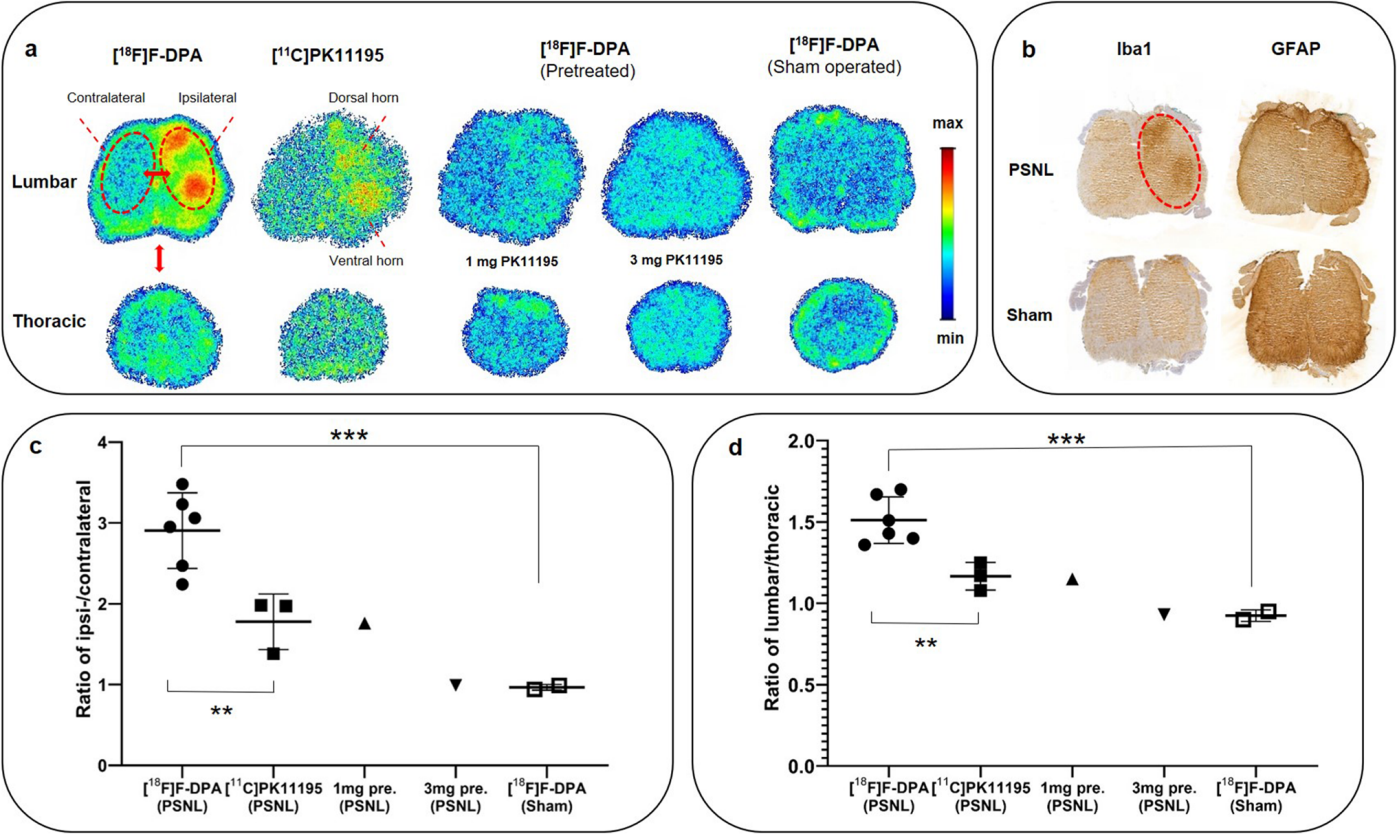
**a** Typical examples of *ex vivo* autoradiography images of [^18^F]F-DPA and [^11^C]PK11195, showing higher accumulation in the dorsal and ventral horns of the spinal cord of PSNL rats. Animals pretreated with 1 mg or 3 mg of the TSPO ligand PK11195 (i.p. injection 30 min prior [^18^F]F-DPA) showed a 70% and 100% reduction in the tracer uptake, respectively. Sham operated animals did not accumulate [^18^F]F-DPA in the spinal cord. **b** Immunohistochemical staining revealed upregulated microglia (Iba1) in the dorsal and ventral horns of the spinal cord that coincided with the [^18^F]F-DPA uptake, whereas the astrocytes (GFAP) levels remained unaffected. **c** Ipsi-/contralateral ratios and (**c**) lumbar/thoracic ratios clearly demonstrate the superiority of [^18^F]F-DPA compared to [^11^C]PK11195. Data are presented as mean ± SD, ***p < 0.0001, **p < 0.01

Typical autoradiograms of [^18^F]F-DPA and [^11^C]PK11195 uptake in the lumbar and thoracic spinal cords of PSNL rats are shown in Fig. 3a. The preferential accumulation of radioactivity was apparent in the ipsilateral dorsal and ventral horns of the lumbar spinal cord. Immunohistochemical staining against Iba1 of the slices demonstrated increased microglial activation in the lumbar area, whereas astrocyte (GFAP) activation was negligible. When comparing the ipsi- and contralateral sides of the lumbar spinal cord, significantly higher (*p* < 0.01) PSL/mm^2^ ratios (2.91 ± 0.47) for [^18^F]F-DPA were detected compared with 1.78 ± 0.34 for [^11^C]PK11195 (Fig. 3b). For regions that covered the entire spinal cord in the lumbar and thoracic regions, the lumbar/thoracic PSL/mm^2^ ratios were 1.51 ± 0.14 and 1.17 ± 0.09, respectively, corresponding to [^18^F]F-DPA and [^11^C]PK11195 (Fig. 3c), and were significantly different (*p* < 0.01). PSNL rats premedicated with 1 mg of PK11195 reduced the [^18^F]F-DPA uptake by 70% in the lumbar area of the spinal cord (lumbar/thoracic ratio; 1.15), whereas a complete blockade was achieved with a 3-mg dose of PK11195 (lumbar/thoracic ratio; 0.93) (Fig. 3). Sham animals did not show any specific accumulation of [^18^F]F-DPA in the spinal cord (Fig. 3).

### Biodistribution of [^18^F]F-DPA

The *ex vivo* biodistribution of [^18^F]F-DPA in tissues is shown in Table 2. Notably, we found a twofold higher tracer uptake in the vertebral body, compared to bone from the skull.

**Table 2.** *Ex vivo* biodistribution of [^18^F]F-DPA (40 min post-injection) in a few tissues from PSNL animals. Data (n = 6/tissue) are expressed as mean ± SD

Tissue	SUV
Blood	0.17 ± 0.02
Thoracic spinal cord	0.27 ± 0.09
Lumbar spinal cord	0.23 ± 0.07
Lumbar vertebra	0.78 ± 0.20
Skull	0.41 ± 0.17

### *Ex Vivo* Imaging of the PSNL Model Spinal Cord

**Fig. 4.**
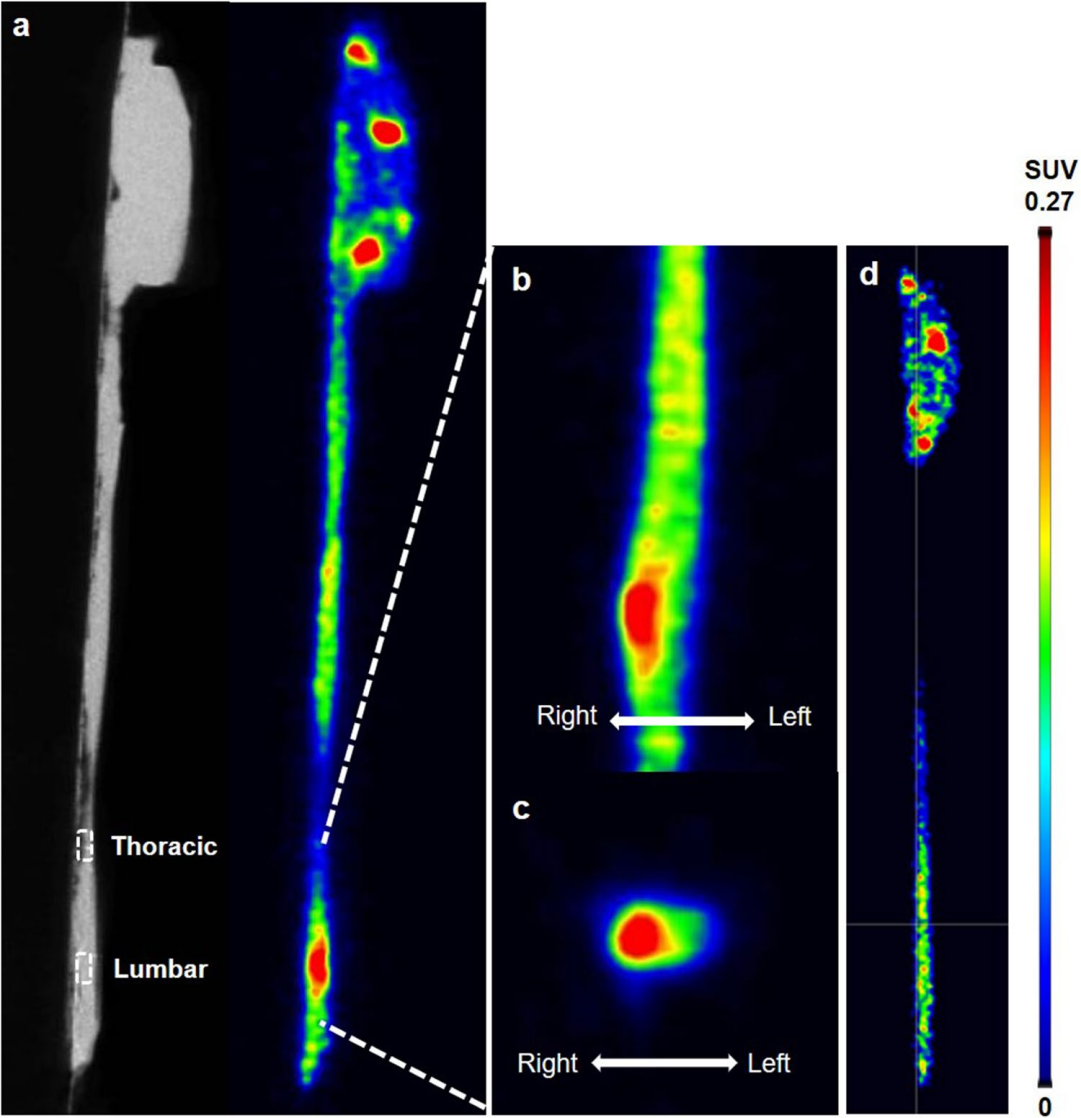
*Ex vivo* PET/CT images of the brain and spinal cord removed from a PSNL and a sham-operated animal at 40 min post-injection of [^18^F]F-DPA. (**a**) Sagittal view of the CT (left) and PET (right) images of a PSNL animal, illustrating the location of the lesion (lumbar) and reference (thoracic) regions. Enlarged coronal (**b**) and transaxial (**c**) view of [^18^F]F-DPA images, indicating unilateral right-side accumulation of [^18^F]F-DPA in the spinal cord. (**d**) Sagittal view of the PET image of a sham-operated animal

*Ex vivo* [^18^F]F-DPA scans of the removed spinal cords showed increased accumulation of [^18^F]F-DPA in the inflammation site (Fig. 4a), which furthermore was located on the right side of the lumbar spinal cord (Fig. 4b–c). Sham animals did not accumulate [^18^F]F-DPA in the lumbar area of the spinal cord (Fig. 4d). The averaged SUVs and SUVs at the peak in the lumbar spinal cord were 0.20 ± 0.02 and 0.38 ± 0.05, respectively. Lumbar/thoracic ratios of SUVs were 1.43 ± 0.002 and 2.69 ± 0.15, corresponding respectively to the averaged SUVs and SUVs at the peak.

### *In Vivo* PET Imaging

**Fig. 5.**
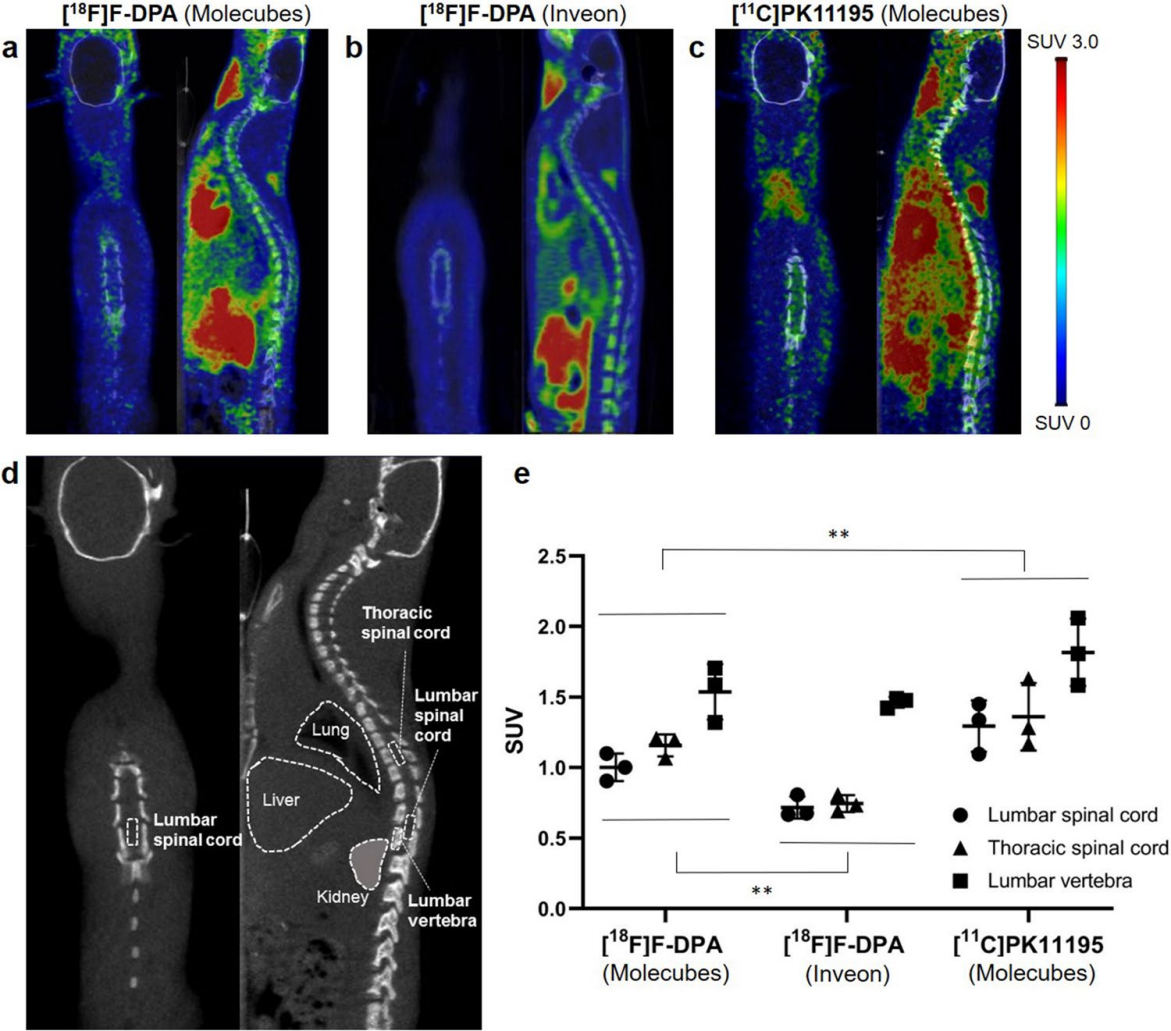
Illustrative PET/CT images summed over 40–60 min post-injection of PSNL rats scanned by (**a**) [^18^F]F-DPA in Molecubes, (**b**) [^18^F]F-DPA in Inveon, and (**c**) [^11^C]PK11195 in Molecubes. (**d**) The location of the analyzed VOIs (dotted boxes) are shown on a CT image. The location of organs that might cause the spillover signal to the spinal cord is also shown. (**e**) Uptake of [^18^F]F-DPA and [^11^C]PK11195 expressed as SUVs in the inflammation site (lumbar spinal cord), reference (thoracic spinal cord), and bone (lumbar vertebra) areas. Significantly higher SUVs (**p < 0.01) were seen for [^11^C]PK11195 in Molecubes compared with SUVs with [^18^F]F-DPA. Significantly higher [^18^F]F-DPA SUVs (**p < 0.01) also were achieved with Molecubes compared with Inveon. *n* = 3/group

Typical whole-body [^18^F]F-DPA and [^11^C]PK11195 images are shown in Fig. 5a–c. The anatomical locations of the analyzed areas are shown in Fig. 5d. [^18^F]F-DPA images were similar between the Molecubes (Fig. 5a) and Inveon scanners (Fig. 5b), but the Molecubes produced images with higher spatial resolution and increased statistical noise. [^18^F]F-DPA was accumulated in the vertebral column and its surrounding regions, and even higher accumulations were seen in further ventral regions. [^11^C]PK11195 with the Molecubes (Fig. 5c) yielded similar images, but much higher radioactivity could be seen in the vertebrae and surrounding regions. Higher background noise was more apparent in the spinal cord region with [^11^C]PK11195 than with [^18^F]F-DPA.

The comparison of SUVs in the inflammation site (lumbar spinal cord), reference (thoracic spinal cord), and lumbar vertebra (vertebra bone including bone marrow) areas with each tracer and scanner is shown in Fig. 5e. The [^18^F]F-DPA SUVs in the lumbar and thoracic regions of the spinal cord were 1.00 ± 0.10 and 1.16 ± 0.08, respectively, using the Molecubes scanner, and 0.72 ± 0.08 and 0.75 ± 0.06, respectively, using the Inveon scanner. The [^18^F]F-DPA SUV value in the inflammation site was approximately 70% and 50% lower compared with that in the vertebral body achieved with the Molecubes and Inveon, respectively. This tendency was also apparent in SUVs as assessed with the [^11^C]PK11195 using the Molecubes scanner (Fig. 5e). It should be noted that no significant difference was found between the lumbar and thoracic regions of the spinal cord in any of the three sets of *in vivo* PET images.

## Discussion

In the current study, we have shown the utility of [^18^F]F-DPA for displaying an inflammation site in the lumbar spinal cord after partial sciatic nerve ligation using *ex vivo* autoradiography. A higher lesion-to-background uptake was measured by *ex vivo* autoradiography with [^18^F]F-DPA compared with [^11^C]PK11195. The [^18^F]F-DPA accumulation was later confirmed by immunohistochemical stainings to coincide with microglia activation in the lumbar spinal cord region. We were also able to block the [^18^F]F-DPA uptake in the inflammation site with the TSPO-selective ligand PK11195. A PK11195 dose of 1 mg reduced the [^18^F]F-DPA uptake by 70%, which is in line with our previous studies in brain and tumors [12, 15]. Increasing the PK11195 dose to 3 mg resulted in a complete blockade of the tracer uptake. Unfortunately, the *A*_m_ was lower in the latter blocking study because it was done with another synthesis device due to temporary but long-lasting facility closure. Although the injected mass of [^18^F]F-DPA was 27.7 µg/kg, we have previously shown that an injected mass of 38 µg/kg of [^18^F]F-DPA can detect upregulated microglia in brain regions of APP/PS1-21 mice [24]. Our results from the *ex vivo* PET imaging study, in which the whole spinal cord and brain from PSNL rats were removed and scanned by Molecubes, also showed clear [^18^F]F-DPA accumulation in the inflammation site of the spinal cord. The maximum peak SUV ratio of lumbar-to-thoracic was 2.69, which was high enough to be visualized as a hot spot in the *ex vivo* PET image. The lumbar-to-thoracic ratio of the averaged SUVs (1.43 ± 0.002) inside the VOIs observed in the *ex vivo* PET image was consistent with the result with *ex vivo* autoradiography (1.51 ± 0.14). The right-to-left asymmetry caused by unilateral sciatic nerve ligation was also visible in the coronal and transaxial images of the *ex vivo* PET, as well as the *ex vivo* autoradiography. These results demonstrated the suitability of the PET imaging technique using the Molecubes scanner to detect the inflammation site in the removed spinal cord in the rat PSNL model.

It should be noted, however, that the present *in vivo* PET did not visualize increased uptake of [^18^F]F-DPA in the inflammation site in this model, either with the Molecubes or Inveon scanner. These findings were not consistent with Imamoto et al.’s report [10]. The lumbar/thoracic ratio from VOIs also showed no significant increase. The contributory factor of this result was the low averaged SUVs in the lumbar spinal cord VOI as shown in the *ex vivo* PET imaging and biodistribution studies (0.20 ± 0.02 and 0.23 ± 0.07, respectively). In contrast, the *in vivo* PET yielded considerably higher SUVs in the inflammation site, which were even higher with Molecubes (1.00 ± 0.10) than Inveon (0.72 ± 0.08). In other words, significant background noise compromised the *in vivo* imaging over the entire spinal cord region. The *in vivo* PET imaging using [^11^C]PK11195 also did not allow detection of activated microglia expression and showed a higher background signal than [^18^F]F-DPA. The reason for the discrepancy from Imamoto et al.’s findings [10] is unknown, but our results were rather reproducible among animals and consistent across *in vivo* PET imaging, *ex vivo* PET imaging, autoradiography, and biodistribution study. Further systematic studies are needed.

As indicated by the *in vivo* PET images presented here, greater accumulation was apparent in the vertebral body compared with the spinal cord with both [^18^F]F-DPA and [^11^C]PK11195. Previous reports [25, 26] have suggested that high uptake of TSPO PET tracers in the bone marrow (part of the vertebral body) is associated with the hematopoietic stem cells residing in the medulla. Gent et al. [26] reported that bone/bone marrow uptake was higher with [^11^C]PK11195 compared with [^11^C]DPA-713 and [^18^F]DPA-714. We found no significant differences in SUVs between [^18^F]F-DPA and [^11^C]PK11195 in the vertebrae, and have previously shown very low defluorination of [^18^F]F-DPA [11], indicating that the uptake originates from the bone marrow. In line with the above, we found a twofold higher [^18^F]F-DPA uptake in vertebrae compared to that seen in bone from the skull. This does not, however, fully explain the fivefold discrepancy in results between the *ex vivo* and *in vivo* findings.

In general, quantitation of a radioactive object in an area surrounded by higher radioactivity can be challenging, particularly when the object is small and/or the radioactivity concentration is low relative to the surroundings [27]. It is worth noting that SUV values in both lumbar and thoracic spinal cord regions obtained from *in vivo* PET are higher when imaged with Molecubes as compared with Inveon, although the former PET scanner has higher spatial resolution than the latter. This suggested that the low-frequency background noise is present along the spinal cord, which is caused by radioactivity distributed in entire parts of the body in addition to the vertebral marrow. Scatter could be one of the factors, because of the smaller ring diameter in Molecubes (76 mm) than Inveon (161 mm) [18]. A recent study [28] has demonstrated that each part of the PET scanner can be a source of scattering in clinical PET systems. This phenomenon is likely more crucial in small animal PET scanners which have a smaller diameter of the detector ring. The high random rate might also be a source of low-frequency bias, which should be more enhanced because the PET imaging has been carried out with ~ 10 MBq of injection dose, which is just below the limit for the counting rate of the Molecubes. An additional factor could be attributed to the performance of the reconstruction software [29]. Even though these are extremely important issues, we defined them beyond the scope of this study.

Although the present study demonstrated that [^18^F]F-DPA has a significantly higher affinity to the small inflammation site in the spinal cord as compared to [^11^C]PK11195, an application to the *in vivo* PET imaging is still limited in the rat PSNL model, attributed to the low-frequency background noise in the spinal cord region caused by several potential physical factors. This may also be the case in clinical application. A large inflammation site in the spinal cord might be visible with *in vivo* [^18^F]F-DPA as demonstrated in the rat model of spinal cord injury (SCI) in our institution [14]; however, small lesion size and a low-grade inflammation might still limit diagnostic usage for neuropathic pain patients. A systematic study to identify the source of the background noise is of paramount importance and such work would encourage efforts to overcome the limitations for enabling further sensitive *in vivo* imaging of small lesions in clinical and preclinical PET studies.

## Conclusions

In this study, [^18^F]F-DPA was superior to [^11^C]PK11195 for detecting spinal cord TSPO expression in a PSNL rat model using *ex vivo* autoradiography and demonstrated clear accumulation on *ex vivo* PET imaging of the removed spinal cord. Despite the high spatial resolution and sensitivity of the Molecubes scanner and the higher TSPO specificity of [^18^F]F-DPA relative to [^11^C]PK11195, we could not detect the inflammation site in the PSNL model *in vivo*. Because of the consistent results among *in vivo* PET imaging, *ex vivo* PET imaging, autoradiography, and biodistribution studies, this is considered attributed to the small lesion size and insufficient SUVs relative to the low-frequency background noise in the restricted spinal cord region, due to several physical factors, in addition to spillover from the vertebral marrow.
